# Still spurious: A comment on attempts to revive cognitive ability tilts

**DOI:** 10.1371/journal.pone.0326486

**Published:** 2025-10-24

**Authors:** Kimmo Sorjonen, Bo Melin, Gustav Nilsonne

**Affiliations:** 1 Department of Clinical Neuroscience, Karolinska Institutet, Stockholm, Sweden; 2 Department of Psychology, Stockholm University, Stockholm, Sweden; University of Lagos Faculty of Engineering, NIGERIA

## Abstract

Ability tilts are within-individual differences between scores on two ability measures, e.g., math – verbal ability. We have shown in a series of reports that correlations between tilts and other variables are spurious consequences of associations to the constituent variables. Recently, Woodley of Menie et al. suggested that findings of incremental validity of tilts, over and above one of the constituent variables, refuted our claims of spuriousness. However, we show here that incremental validity of tilts are spurious consequences of incremental validity of the constituent variables. Moreover, Woodley of Menie et al. presented new results where so-called “tilt super-residuals” were attributable to shared environmental factors and they concluded that this finding confirmed a hypothesis that individuals specialize with respect to cognitive niches as an effort to adapt to stable environmental factors, alternatively do not specialize in the case of an unstable environment. However, we show that variance on “super-residualized” tilts attributable to shared environmental factors is a spurious consequence of adjusting for a variable (e.g., age) that is identical within twin couples. In summary, findings involving ability tilts still appear to be spurious.

## Introduction

Ability tilts are within-individual differences between scores on two ability measures, e.g., math – verbal ability. Studies, mainly by Coyle and colleagues, have reported correlations between ability tilts and other measures of the constituent abilities, e.g., positive correlations between a math-verbal tilt and other measures of math ability than the one used to estimate the tilt, and negative correlation between the tilt and other measures of verbal ability [1–8]. Coyle and colleagues have concluded that such tilt correlations support investment theories, suggesting that individuals have invested different amounts of time and effort into cultivating one of the abilities, e.g., math ability, at the expense of the other ability, e.g., verbal ability [6–9].

However, we have challenged these explanations based on differential investment, with reference to the mathematical fact that the expected correlation between a X-Y difference (tilt) and a third variable Z is a function of the difference between the correlation between X and Z (*r*_*XZ*_) and the correlation between Y and Z (*r*_*YZ*_) (Eq. 1) [10]. Consequently, positive correlations between, for example, a math-verbal tilt and other measures of math ability arise spuriously, since different measures of the same ability tend to be more strongly correlated than measures of different abilities [11,12]. Anyone claiming that tilt correlations support differential investment should then, for the sake of consistency, also assume, for example, that some U.S. states have invested more in births while other states have invested more in deaths [12] and that some individuals have invested more in a large head at the expense of verbal ability and vice versa [13]. We find mathematical explanations based on differences between correlations (Eq. 1) more likely.


$$E(r_{X-Y,\;Z})=\frac{r_{XZ}-r_{YZ}}{\sqrt{\text{2}(\text{1}-r_{XY})}}$$ Eq. 1

Coyle et al. [14] analyzed twin data and presented results indicating that ability tilts were genetically heritable. This would presumably mean that the human genome codes for differences between cognitive abilities, e.g., math-verbal, in addition to the constituent abilities, e.g., math and verbal. However, we have shown that genetic heritability of ability tilts are spurious consequences of heritability of the constituent variables. The heritability of a X-Y tilt tends to equal the mean of the heritability of X and the heritability of Y. Anyone assuming that heritability of ability tilts are genuine, i.e., non-spurious, should, then, also assume, for example, that the human genome codes for a difference between spatial ability and nose length [13]. Coyle et al. [14] also found a high degree of variance in tilts to be attributable to non-shared environmental factors (i.e., environmentality), which they suggested to be a consequence of niche-picking. However, we found the environmentality of tilts to be a spurious consequence of the environmentality of the constituent variables. Anyone using niche-picking as an explanation should, then, use the same explanation for the environmentality of, for example, a spatial ability – head circumference tilt [13]. We do not endorse this explanation.

Recently, Woodley of Menie et al. [1], the same authors who contributed to Coyle et al. [14], replied to our critique by referencing findings from Kato and Scherbaum [15] and presenting their own new results, which they argue contradict our assertions regarding the spurious nature of the associations and heritability of ability tilts as mere consequences of the associations and heritability of the constituent variables. Here, we address the points raised by Woodley of Menie et al.

In summary, we have previously questioned research on ability tilts and shown that certain proposed correlations with ability tilts are due to statistical artifacts. Woodley of Menie et al. have responded by citing previous research and presenting new findings that they claim challenge our conclusions about the spurious associations and heritability of ability tilts. Here, we address their arguments directly and contribute further empirical evidence suggesting that statistical artifacts can explain previously reported correlations to and heritability of ability tilts.

### Data and analyses

The present comment, like our previous study [13] and challenged studies by Coyle et al. [14] and Woodley of Menie et al. [1], used data from the Georgia Twin Study [16]. Data were collected from 108 monozygotic and 130 dizygotic twin couples (*N* = 476). Data included the four Primary Mental Abilities Tests Verbal meaning, Number facility, Reasoning, and Spatial relations. Data also included biometric measures of face length and nose length (among other measures not analyzed here). We added two random normal variables to the data and all cognitive and biometric measures were standardized (*M* = 0, *SD* = 1) before calculations of tilts and further analyses.

We analyzed data with ordinary least squares (OLS) regression analyses, factor analyses, and behavior-genetic ACE models, which estimate the percentage of variance in outcomes that can be attributed to genetic heritability (A), shared environmental factors (C), and non-shared environmental factors (E). See below, under the separate headings, for more information on how the analyses were conducted. Analyses were conducted with R 4.4.0 statistical software [17] employing the mets [18], psych [19], paran [20], and osfr [21] packages. Data and the analytic script are available at the Open Science Framework at https://osf.io/w4jad/.

### Effects of residualized tilts in Kato and Scherbaum (2023)

Woodley of Menie et al. [1] referred to Kato and Scherbaum [15], who found incremental validity, i.e., an increase in explained variance (*R*^*2*^), of job-relevant ability tilts over and above what could be explained by *g* (general cognitive ability) and one of the constituent abilities when predicting job performance. According to Woodley of Menie et al., this incremental validity contradicted our claims that associations involving tilts are spurious consequences of associations involving the constituent variables. However, it must be noted that Kato and Scherbaum did not adjust for both of the constituent variables X and Y when estimating incremental validity of the X-Y tilt. To do so would have been impossible, because if both X and Y are kept constant there is no variance in X-Y (for all individuals the value on the X-Y tilt would, by definition, be exactly the same as the difference between their value on X and on Y). Instead, Kato and Scherbaum adjusted only for one of the constituent variables. However, some algebra reveals that:


$$\;\begin{array}{cc} \text{if } & \text{Z }=\;b_{\text{1}}\text{X }+\;b_{\text{2}}(\text{X}-\text{Y}) \end{array}\;$$ Eq. 2

$$\begin{array}{cc} \text{then} & \text{Z }=\;b_{\text{1}}\text{X }+\;b_{\text{2}}\text{X }-\;{\text{b}}_{\text{2}}\text{Y} \end{array}\;$$ Eq. 3

$$\;\begin{array}{cc} \text{and} & \text{Z }=\;(b_{\text{1}}\;+\;b_{\text{2}})\text{X }-\;b_{\text{2}}\text{Y} \end{array}$$ Eq. 4

This means that predicting an outcome from a tilt and one of the constituent variables (Eq. 2) is equivalent to predicting the outcome from the two constituent variables (Eq. 4). The predictive validity of the two models would be exactly the same. Moreover, the coefficients in the two models (*b*_*1*_ and *b*_*2*_) would be perfect functions of each other. This fact would not change if adding a third variable, e.g., *g*, to Eq. 2.

For all 4 × 3 × 2 = 24 combinations of the four ability measures in the Georgia Twin Study as Z, X, and Y variables, we fitted the following three models to data: (1) Z = *b*_*1*_X + *b*_*2*_Y; (2) Z = *b*_*1*_X + *b*_*2*_(X-Y); (3) Z = *b*_*1*_X + *b*_*2*_(Y-X). The analyses revealed, as predicted above, that: (1) The predictive validity of the models were identical (*R*^*2*^ in columns 3, 6, and 9 in Table 1 are identical); (2) The coefficient for X in models 2 and 3 was the sum of the coefficients for X and Y in model 1 (values in columns 4 and 7 in Table 1 are equal to values in column 1 + values in column 2); (3) The coefficient for the X-Y tilt in model 2 was equal to the negation of the coefficient for Y in model 1 (values in column 5 in Table 1 equal −1 × values in column 2); (4) The coefficient for the Y-X tilt in model 3 was equal to the coefficient for Y in model 1 (values in column 8 in Table 1 are identical to values in column 2).

**Table 1:** Coefficients for X, Y, X-Y, and Y-X, and amount of explained variance (*R*^*2*^), when predicting Z, for all combinations of four cognitive measures as Z, X, and Y variables, respectively. Data from the Georgia Twin Study (*N* = 476).

			Z = *b*_*1*_X + *b*_*2*_Y			Z = *b*_*1*_X + *b*_*2*_(X-Y)			Z = *b*_*1*_X + *b*_*2*_(Y-X)		
Z	X	Y	1.*b*_*1*_	2.*b*_*2*_	3.*R*^*2*^	4.*b*_1_	5.*b*_*2*_	6.*R*^*2*^	7.*b*_*1*_	8.*b*_*2*_	9.*R*^*2*^
VE	NU	RE	0.26	0.52	0.52	0.77	−0.52	0.52	0.77	0.52	0.52
VE	NU	SP	0.50	0.22	0.42	0.72	−0.22	0.42	0.72	0.22	0.42
VE	RE	NU	0.52	0.26	0.52	0.77	−0.26	0.52	0.77	0.26	0.52
VE	RE	SP	0.63	0.10	0.49	0.74	−0.10	0.49	0.74	0.10	0.49
VE	SP	NU	0.22	0.50	0.42	0.72	−0.50	0.42	0.72	0.50	0.42
VE	SP	RE	0.10	0.63	0.49	0.74	−0.63	0.49	0.74	0.63	0.49
NU	VE	RE	0.24	0.55	0.54	0.79	−0.55	0.54	0.79	0.55	0.54
NU	VE	SP	0.47	0.32	0.47	0.79	−0.32	0.47	0.79	0.32	0.47
NU	RE	VE	0.55	0.24	0.54	0.79	−0.24	0.54	0.79	0.24	0.54
NU	RE	SP	0.61	0.18	0.53	0.78	−0.18	0.53	0.78	0.18	0.53
NU	SP	VE	0.32	0.47	0.47	0.79	−0.47	0.47	0.79	0.47	0.47
NU	SP	RE	0.18	0.61	0.53	0.78	−0.61	0.53	0.78	0.61	0.53
RE	VE	NU	0.41	0.46	0.62	0.87	−0.46	0.62	0.87	0.46	0.62
RE	VE	SP	0.52	0.36	0.59	0.88	−0.36	0.59	0.88	0.36	0.59
RE	NU	VE	0.46	0.41	0.62	0.87	−0.41	0.62	0.87	0.41	0.62
RE	NU	SP	0.54	0.32	0.58	0.86	−0.32	0.58	0.86	0.32	0.58
RE	SP	VE	0.36	0.52	0.59	0.88	−0.52	0.59	0.88	0.52	0.59
RE	SP	NU	0.32	0.54	0.58	0.86	−0.54	0.58	0.86	0.54	0.58
SP	VE	NU	0.25	0.40	0.34	0.65	−0.40	0.34	0.65	0.40	0.34
SP	VE	RE	0.13	0.53	0.39	0.66	−0.53	0.39	0.66	0.53	0.39
SP	NU	VE	0.40	0.25	0.34	0.65	−0.25	0.34	0.65	0.25	0.34
SP	NU	RE	0.22	0.46	0.41	0.68	−0.46	0.41	0.68	0.46	0.41
SP	RE	VE	0.53	0.13	0.39	0.66	−0.13	0.39	0.66	0.13	0.39
SP	RE	NU	0.46	0.22	0.41	0.68	−0.22	0.41	0.68	0.22	0.41

Note: VE = Verbal meaning, NU = Number facility, RE = Reasoning, SP = Spatial relations.

In summary, these findings showed that incremental validity and regression coefficients of tilts when adjusting for one of the constituent variables are spurious consequences of incremental validity and coefficients of the constituent variables. Consequently, the claim by Woodley of Menie et al. [1], that effects of residualized tilts presented by Kato and Scherbaum [15] contradicted our claims that associations involving tilts are spurious consequences of associations involving the constituent variables, can be questioned.

### The novel “only within the same factor” argument

Woodley of Menie et al. [1] presented a novel argument that calculating tilts makes theoretical sense only if the constituent variables load on the same factor. The argument posits, as we understand it, that investing time and effort into achieving one characteristic, e.g., high spatial ability, will be at the expense of another characteristic, e.g., verbal ability, that loads on the same factor but not necessarily at the expense of a characteristic, e.g., a long nose, that does not load on the same factor. Consequently, to estimate a spatial-verbal ability or a nose – face length tilt would be meaningful but not to estimate, as we did [13], a spatial ability – nose length tilt.

We do not agree with this argument. As resources (time, effort, energy) are finite, investment of resources into something, e.g., playing the violin, will always be at the expense of something else that requires the same resources, e.g., long distance running. Both playing the violin and long distance running requires investment of time, effort, and energy and the more of these resources that are invested in one of the activities, the less is available for investment in the other activity. Whether or not the two variables are positively correlated, which is required for them to load positively on the same factor, is not material to the tradeoff. Consequently, if, as Woodley of Menie et al. claim, tradeoffs are at the core of tilts, they should not require the constituent variables to load on the same factor.

Moreover, using differential investment as an argument why some tilts are meaningful, while others are not, appears to presuppose that tilts are due to differential investment. To conclude that associations involving tilts support differential investment [6–9] and then to claim that estimating tilts are meaningful only if they are due to differential investment [1] appears to be a circular argument. It is like claiming that all swans are white and when presented with a black swan to exclaim that observing swans is meaningful only if they are white.

Mathematics is neutral to what values refer to, e.g., 1 + 1 = 2 whether we are counting apples or sheep. As described above, Coyle and colleagues (including Woodley of Menie et al.) [1,6–9,14] claim that tilt correlations between X-Y and Z suggest that levels on X and Y are due to differential investment in one of the variables at the expense of the other, and that genetic heritability/environmentality of X-Y suggests that X-Y is genetically coded over and above X and Y but also due to niche picking and/or specialization. If Coyle and colleagues accept the neutrality of mathematics, they should assert that their conclusions are based on the statistical methods they have used, and not what X and Y refers to. To say that certain conclusions follow from the used methods, but only if X-Y fulfills Coyle and colleagues’ subjective criterion of meaningfulness, is not to accept the neutrality of mathematics. Instead, it is like saying that 1 + 1 = 2, but only if you are counting something cute.

Furthermore, the “only within the same factor” argument would posit that the meaningfulness of a X-Y tilt depends on which other variables happen to be included in the same dataset, even if values on X and Y would be exactly the same. For example, a parallel analysis of eleven variables from the Georgia Twin Study suggested four factors and as spatial ability and nose length loaded on different factors, Woodley of Menie et al. concluded that it would not be meaningful to calculate a spatial ability – nose length tilt. However, when we limited the same dataset to include only the two variables, a parallel analysis suggested (not surprisingly) one factor and although not strongly (factor loading, λ = 0.30), spatial ability and nose length loaded on the same factor (with a similar procedure, the loadings of number ability and head length were λ = 0.54). According to the logic of Woodley of Menie et al., estimating a spatial ability – nose length tilt would be meaningful in the latter (with only these variables in the dataset) but not in the former (with eleven variables in the dataset) case, even if values on spatial ability and nose length would be exactly the same. We believe that this “only within the same factor”-rule is too arbitrary to be useful.

### Heritability of super-residualized tilts in Woodley of Menie et al. (2025)

As mentioned above, it is not possible to estimate the effect of X-Y on Z while adjusting for X and Y. Similarly, it is not possible to estimate the variance composition of X-Y in an ACE model while including X and Y as covariates. Instead, in order to estimate adjusted variance compositions, Woodley of Menie et al. [1] used a procedure that we have not seen before: (1) The X-Y difference between each pair of cognitive measures (standardized) was calculated (Tilt); (2) The tilt was regressed on general cognitive ability (GCA, estimated as a factor score of the four ability scores) and age and the residuals were saved (RES1); (3) RES1 was regressed on all four ability scores and the residuals were saved (RES2, named “tilt super-residuals” by Woodley of Menie et al.); (4) ACE, ADE, and AE models were fitted on RES2.

Of the three models, ACE fitted data (Georgia Twin Study) best and variance in the “super-residualized” tilt was mainly (97%) attributable to shared environmental factors. Woodley of Menie et al. analyzed only one of the six “super-residualized” tilts as they were perfectly correlated with each other (which, in our opinion, should have set off alarm bells). Woodley of Menie et al. concluded that variance attributable to shared environmental factors supported hypotheses of cognitive differentiation-integration efforts, suggesting that individuals specialize with respect to cognitive niches as an effort to adapt to stable environmental factors, alternatively do not specialize in the case of an unstable environment.

However, we suspected that Woodley of Menie et al.’s findings may have been a statistical artifact due to the “super-residualization” procedure. We noted that when estimating RES1 (see above), Woodley of Menie et al. used age as a predictor. Age is the same for both twins in a couple and the correlation between the age of both monozygotic and dizygotic twins is, consequently, equal to unity. Therefore, variance in age attributable to genetic heritability equals 2 × (1–1) = 0 and to shared environmental factors 1–0 = 1 (i.e., 100%). We decided to evaluate if findings by Woodley of Menie et al. could be replicated when replacing age in the “super-residualization” by some other variable that was identical within twin couples, here an arbitrary twin pair ID-number included in the Georgia Twin Study dataset.

For each of the four cognitive measures, face length, nose length, and a random variable, as well as the 21 tilts between these seven variables, we first residualized for either age or pair ID-number and then for the outcome variable or, when the outcome was a tilt, the two constituent variables. This was a slightly simplified version of Woodley of Menie et al.’s procedure, as we did not adjust for general cognitive ability in the first step and only for the two constituent variables, instead for all four cognitive scores, in the second step. We used this simplified version in order to generalize to situations where the outcomes are not measures of cognitive ability. This simplification had no substantial effect on the results and is not material for the argument we are making here.

Variance in all seven “super-residualized” outcome variables (Table 2, rows 1–7) as well as all 21 “super-residualized” tilts (Table 2, rows 8–28) were mainly (> 85%) attributable to shared environmental factors. This was the case irrespective of whether the “super-residualization” had used age (Table 2, column 2) or an arbitrary pair ID-number (Table 2, column 5) in the first step. We estimated an additional tilt between the original random variable and a second random variable with a strong correlation (*r* = 0.81, the size of this correlation is not material for the outcome) with the original random variable. This tilt between random variables adhered to Woodley of Menie et al.’s argument that tilts are meaningful only if the two constituent variables load on the same factor. Similar to the other tilts, variance in the “super-residualized” tilt between these two random variables was mainly attributable to shared environmental factors (Table 2, row 29).

**Table 2:** “Super-residualized” variance in outcome variables and tilts attributable to genetic heritability (A), shared environmental factors (C) and non-shared environmental factors (E) [with 95% CI]. Separately for when the first step in “super-residualization” consisted of adjusting for age (columns 1-3) and an arbitrary twin pair ID-number (columns 4-6).

	Adjusted for Age			Adjusted for Pair-ID		
Outcome	1. A	2. C	3. E	4. A	5. C	6. E
1.VE	.00 [.00;.01]	.99 [.99; 1.0]	.01 [.00;.01]	.01 [.00;.01]	.99 [.98;.99]	.01 [.01;.01]
2.NU	.01 [.00;.01]	.99 [.98;.99]	.01 [.00;.01]	.02 [.00;.03]	.97 [.95;.98]	.02 [.01;.02]
3.RE	.00 [.00;.00]	1.0 [1.0; 1.0]	.00 [.00;.00]	.03 [.01;.05]	.96 [.94;.98]	.01 [.01;.02]
4.SP	.01 [.00;.01]	.99 [.98;.99]	.00 [.00;.01]	.04 [.00;.07]	.93 [.90;.96]	.03 [.02;.04]
5.FL	.01 [.01;.02]	.99 [.98;.99]	.00 [.00;.00]	.01 [.00;.01]	.99 [.99; 1.0]	.00 [.00;.00]
6.NL	.03 [.01;.05]	.95 [.94;.97]	.02 [.01;.02]	.01 [.00;.01]	.99 [.99;.99]	.00 [.00;.00]
7.RA	.00 [.00;.00]	1.0 [1.0; 1.0]	.00 [.00;.00]	.00 [.00;.00]	1.0 [.99; 1.0]	.00 [.00;.00]
8.VE-NU^1^	.01 [.00;.01]	.99 [.98;.99]	.01 [.00;.01]	.02 [.00;.03]	.97 [.96;.98]	.01 [.01;.02]
9.VE-RE^1^	.00 [.00;.00]	.99 [.98;.99]	.01 [.01;.02]	.03 [.01;.04]	.96 [.94;.98]	.01 [.01;.02]
10.VE-SP^1^	.01 [.00;.01]	.99 [.98;.99]	.00 [.00;.01]	.04 [.00;.07]	.93 [.90;.96]	.03 [.02;.04]
11.VE-FL	.01 [.00;.02]	.98 [.98;.99]	.01 [.00;.01]	.05 [.02;.07]	.94 [.91;.96]	.02 [.01;.02]
12.VE-NL	.04 [.02;.06]	.95 [.93;.97]	.01 [.01;.02]	.00 [.00;.01]	.99 [.98;.99]	.01 [.01;.01]
13.VE-RA	.00 [.00;.01]	.99 [.99; 1.0]	.01 [.00;.01]	.00 [−.01;.02]	.98 [.97;.99]	.01 [.01;.02]
14.NU-RE^1^	.02 [.00;.03]	.97 [.95;.98]	.02 [.01;.02]	.03 [.01;.04]	.96 [.95;.98]	.01 [.01;.02]
15.NU-SP^1^	.01 [.00;.02]	.99 [.98;.99]	.01 [.00;.01]	.04 [.01;.07]	.94 [.91;.96]	.03 [.02;.04]
16.NU-FL	.01 [.01;.02]	.98 [.97;.99]	.01 [.00;.01]	.05 [.02;.09]	.92 [.89;.95]	.02 [.02;.03]
17.NU-NL	.04 [.02;.07]	.94 [.91;.96]	.02 [.01;.03]	.02 [.00;.03]	.97 [.95;.98]	.02 [.01;.02]
18.NU-RA	.01 [.00;.01]	.99 [.98;.99]	.01 [.00;.01]	.03 [.01;.05]	.96 [.94;.97]	.01 [.01;.02]
19.RE-SP^1^	.01 [.00;.02]	.98 [.98;.99]	.01 [.00;.01]	.04 [.01;.07]	.94 [.91;.96]	.02 [.01;.03]
20.RE-FL	.01 [.00;.01]	.99 [.99; 1.0]	.00 [.00;.00]	.07 [.04;.10]	.92 [.89;.95]	.01 [.01;.02]
21.RE-NL	.03 [.01;.05]	.95 [.93;.97]	.02 [.01;.02]	.03 [.01;.05]	.96 [.94;.97]	.01 [.01;.02]
22.RE-RA	.00 [.00;.00]	1.0 [1.0; 1.0]	.00 [.00;.00]	.03 [.01;.05]	.96 [.94;.97]	.01 [.01;.02]
23.SP-FL	.01 [.00;.02]	.98 [.98;.99]	.01 [.00;.01]	.09 [.04;.14]	.87 [.82;.92]	.04 [.02;.05]
24.SP-NL	.03 [.01;.05]	.95 [.93;.97]	.02 [.01;.03]	.04 [.00;.08]	.93 [.89;.96]	.03 [.02;.04]
25.SP-RA	.01 [.00;.01]	.99 [.98;.99]	.01 [.00;.01]	.04 [.01;.08]	.93 [.89;.96]	.03 [.02;.05]
26.FL-NL^1^	.04 [.02;.06]	.95 [.93;.97]	.01 [.01;.02]	.01 [.00;.01]	.99 [.98;.99]	.01 [.00;.01]
27.FL-RA	.01 [.01;.02]	.99 [.98;.99]	.00 [.00;.00]	.01 [.00;.01]	.99 [.99;.99]	.00 [.00;.01]
28.NL-RA	.03 [.01;.05]	.95 [.94;.97]	.02 [.01;.02]	.01 [.00;.01]	.99 [.98;.99]	.01 [.00;.01]
29.RA-R2^1^	.00 [.00;.00]	.98 [.97;.98]	.02 [.02;.03]	.00 [.00;.00]	.99 [.99; 1.0]	.00 [.00;.00]

Note: VE = Verbal meaning, NU = Number facility, RE = Reasoning, SP = Spatial relations, FL = face length, NL = nose length, RA = random variable, R2 = a second random variable, 1 Adhered to Woodley of Menie et al.’s argument that constituent variables should load on the same factor.

**Fig 1:**
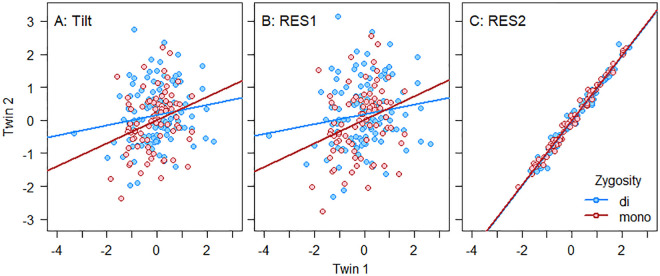
Effect of super-residualization. Correlation between twins’ Verbal – Number ability tilt (A), residual in the tilt not accounted for by age (RES1, B), and residual in RES1 not accounted for by Verbal and Number ability (RES2, C).

The impact of super-residualization is illustrated in Fig 1. The correlation of the Verbal – Number ability tilt was stronger among monozygotic (*r* = 0.306) compared with dizygotic (*r* = 0.153) twins (Fig 1A). The same was true for the residual in the tilt not accounted for by age (RES1, *r* = 0.306 and *r* = 0.153, respectively, Fig 1B). However, the correlation between the residual in RES1 not accounted for by Verbal and Number ability (RES2) was close to unity both among monozygotic (*r* = 0.994) and dizygotic (*r* = 0.991) twins (Fig 1C). Hence, variance in RES2 attributable to genetic heritability was equal to 2 × (0.994–0.991) = 0.006 and to shared environmental factors 0.994–0.006 = 0.988. Adjustment for an arbitrary twin pair ID-number instead of age in the estimation of RES1 would have resulted in almost identical associations (see row 8 in Table 2).

As mentioned above, Woodley of Menie et al. concluded that variance in “tilt super-residuals” attributable to shared environmental factors can be explained by individual specialization with respect to (cognitive) niches. Consequently, according to the logic of Woodley of Menie et al., some participants in the Georgia Twin Study had specialized in receiving a high score on the first random variable by us several decades after birth while others had specialized in receiving a high score on the second random variable, alternatively specialized in achieving a long face at the expense of a long nose or vice versa. We do not find this explanation tenable. We acknowledge that there could be conceptual merit or testable hypotheses worth pursuing in future work based on investment or specialization theories. For example, it has been proposed that among mammals, range size was the selection pressure that acted to increase spatial ability [22–24]. However, as for “super-residualized” variables, tilts or otherwise, we propose that a high degree of variance attributable to shared environmental factors is a spurious consequence of adjusting for a variable (e.g., age or pair-ID) that is identical within twin pairs rather than due to cognitive specialization.

### Summary and conclusions

We have previously concluded that associations involving and genetic heritability of ability tilts (X-Y) are spurious consequences of associations involving and heritability of the constituent variables (X and Y) [11–13]. This means that findings on ability tilts appear to be statistical artifacts. Woodley of Menie et al. [1] claimed that our conclusions faced empirical anomalies (i.e., aberrations), such as findings by Kato and Scherbaum [15], who found incremental validity (i.e., increased explained variance) when adding job-relevant ability tilts as predictors to models where job performance was predicted from one of the constituent variables in the tilt (i.e., adding X-Y to a model where Z was predicted from X). However, here we show that the incremental validity and regression coefficients of tilts are spurious consequences of the incremental validity and regression coefficients of the constituent variables.

Moreover, Woodley of Menie et al. presented results suggesting that variance in residualized tilts were mainly attributable to shared environmental factors and concluded that these findings could be explained by individual specialization with respect to cognitive niches. However, here we show that these findings were spurious consequences (statistical artifacts) of Woodley of Menie et al.’s procedure of “super-residualization”, where tilts were residualized for the constituent variables as well as a variable (age) that was identical within twin couples. Furthermore, if, as concluded by Woodley of Menie et al., “super-residualized” tilts attributable to shared environmental factors confirm that tilts are due to specialization, we should, for consistency, also conclude that some participants in the Georgia Twin Study had specialized in attaining a high score on a first random variable from us several decades after birth while others had specialized in attaining a high score on a second random variable. We find an explanation based on statistical artifacts and spuriousness more likely and conclude that the present results add to a growing body of research showing that findings involving ability tilts are spurious.
